# Successful Treatment of a Thyroid Abscess in a Pediatric Patient: A Case Report

**DOI:** 10.7759/cureus.57428

**Published:** 2024-04-01

**Authors:** Josiah Williams, Azwan Halim Abdul Wahab, Wan Ishlah Leman

**Affiliations:** 1 Department of Otolaryngology-Head and Neck Surgery, International Islamic University Malaysia, Kuantan, MYS

**Keywords:** investigations, case, management, pediatrics, thyroid abscess

## Abstract

Thyroid abscess, or acute suppurative thyroiditis (AST), is an exceedingly rare condition, particularly in the pediatric population. It often results from congenital anomalies or is secondary to infections. Despite its rarity, prompt diagnosis and management are crucial to prevent serious complications. We report a case of a five-year-old girl with no significant medical history who presented with a two-week history of anterior neck swelling, odynophagia, fever, and leukocytosis. Notably, the patient did not exhibit symptoms of thyroid dysfunction. Initial treatment with antibiotics for a suspected bacterial infection at a private clinic did not lead to improvement. Ultrasound and computed tomography scans revealed a multiloculated abscess within the left thyroid lobe. The patient underwent successful incision and drainage, supported by antibiotic therapy, resulting in a full recovery without complications. Imaging studies played a critical role in diagnosing and guiding the management of this condition. Thyroid abscess, though rare, should be part of the differential diagnosis for pediatric patients presenting with acute neck swelling, fever, and pain. Early diagnosis and appropriate management, typically involving surgical drainage and antibiotics, are essential for a favorable outcome.

## Introduction

The thyroid gland consists of two symmetrical lobes located on either side of the trachea, connected by an isthmus. It is surrounded by a capsule that originates from the pre-tracheal fascia. This gland features both a true and a false capsule; the former being a condensation of connective tissue and the latter formed by the pre-tracheal fascia. Venous plexuses are situated between these two capsules. The thyroid is a highly vascular organ, receiving blood from the superior and inferior thyroid arteries and, in 3% of individuals, from the thyroid ima artery [1]. Venous drainage is through the superior, middle, and inferior thyroid veins. The gland's high vascularity, encapsulation, and the presence of iodine and peroxidase make it resistant to infections [2]. Pediatric thyroid abscesses often result from a pyriform sinus fistula [3,4]. This report describes the case of a five-year-old child with a thyroid abscess and outlines the management approach.

## Case presentation

A five-year-old girl, previously healthy with no significant postnatal history, was brought to the emergency department due to an anterior neck swelling present for two weeks, accompanied by fever and pain at the swelling site. The swelling, initially measuring 1.0 cm x 1.0 cm, had grown to approximately 2.0 cm x 2.0 cm over two weeks. The swelling persisted despite receiving a week-long course of antibiotics targeting gram-positive bacteria at a private clinic. The child also experienced odynophagia and restricted neck movement to the left, but no dysphagia, stridor, hoarseness, drooling, or thyroid dysfunction symptoms were reported.

Upon examination in the emergency department, the child had stable vitals, except for a low-grade fever of 37.5°C. She appeared active and not septic and her voice was assessed as normal. The neck examination revealed a firm, tender swelling measuring 3.0 cm x 2.0 cm at the left level two region, with no skin changes, visible punctum, or attachment to underlying structures, and it moved with swallowing. No cervical lymphadenopathy was detected. An awake flexible fiberoptic nasopharyngolaryngoscopy showed no abnormalities in the pharyngeal wall or supraglottic structures, including the vocal cords.

**Figure 1:**
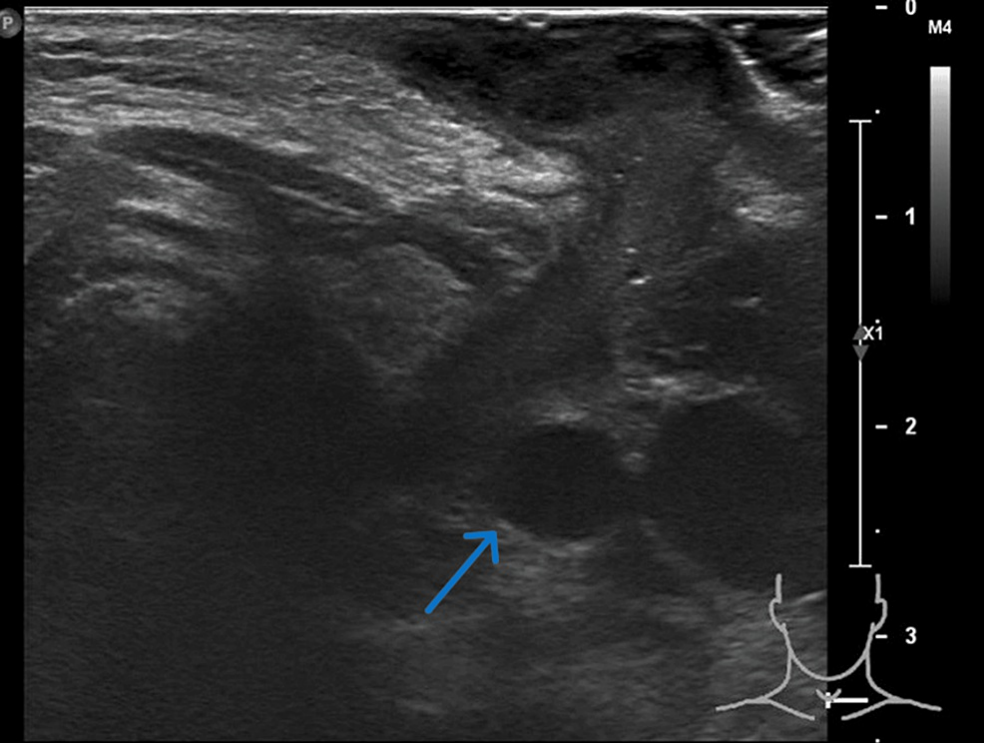
Ultrasonography image of the neck Ultrasound of the neck shows a collection within the left thyroid lobe (blue arrow).

Laboratory tests revealed a white cell count of 12 x 10^9^/L, with a predominance of neutrophils. An AP view neck X-ray indicated a soft tissue density causing tracheal deviation to the right. A neck ultrasound revealed a large heterogeneous hypoechoic lesion within the left thyroid lobe, measuring approximately 1.5 cm x 3.0 cm x 2.4 cm (anteroposterior x width x craniocaudal, respectively (Figure 1).

**Figure 2:**
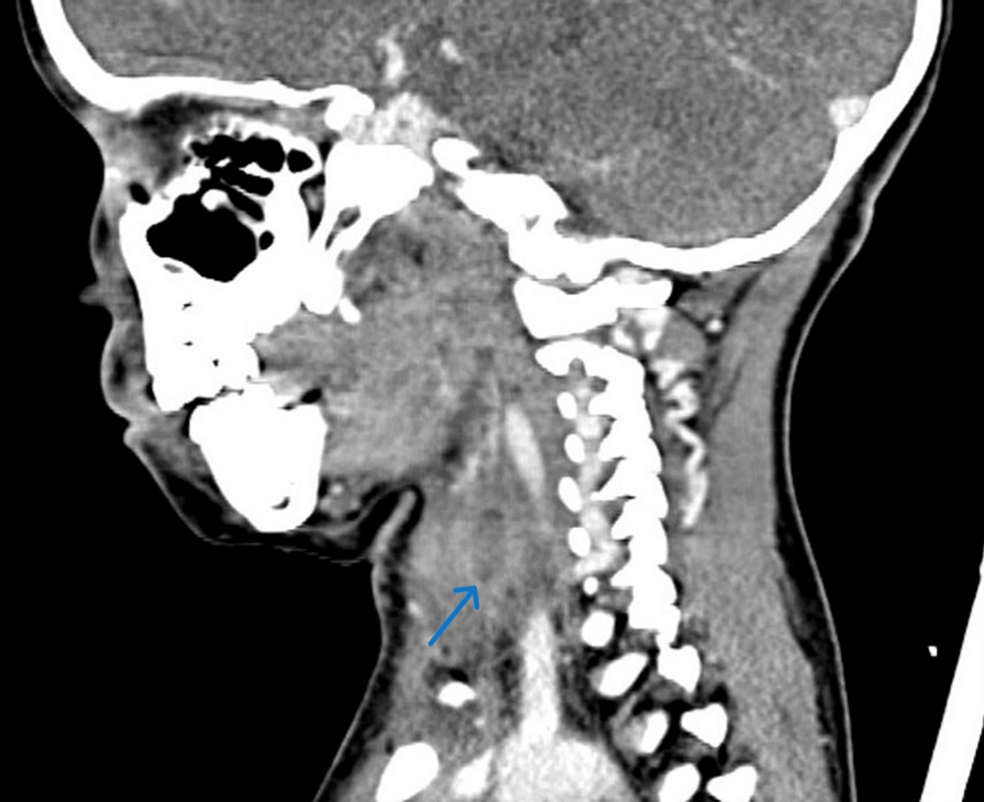
Sagittal view of contrast-enhanced computed tomography of the neck

Contrast-enhanced computed tomography (CT) of the neck identified a multiloculated enhancing collection within the left thyroid lobe suggestive of an abscess, with mass effect on the trachea and extension into the retropharyngeal space (Figure 2). Based on these findings, a diagnosis of thyroid abscess was made, leading to a decision to perform incision and drainage.

**Figure 3:**
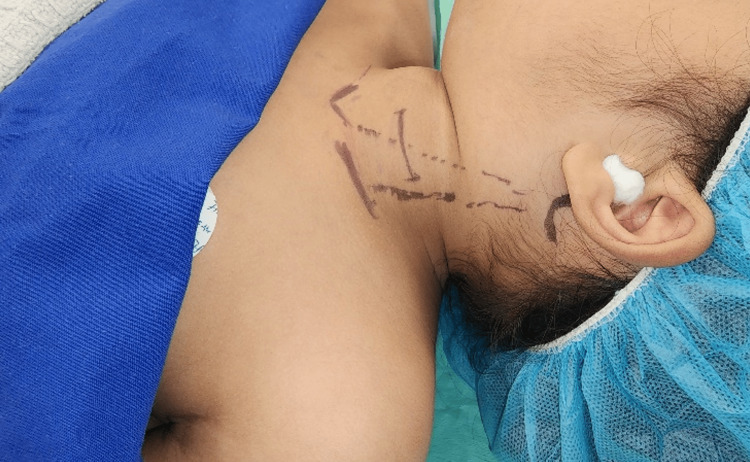
Perioperative photograph of surgical site marking

**Figure 4:**
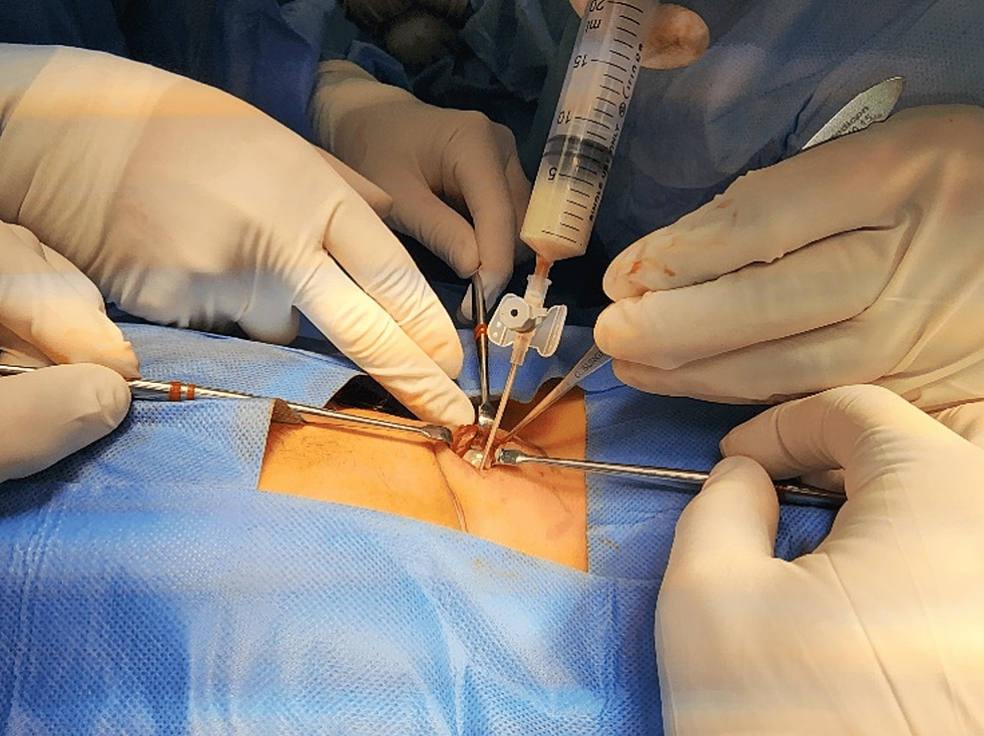
Perioperative photograph of abscess aspiration from the left upper thyroid lobe

**Figure 5:**
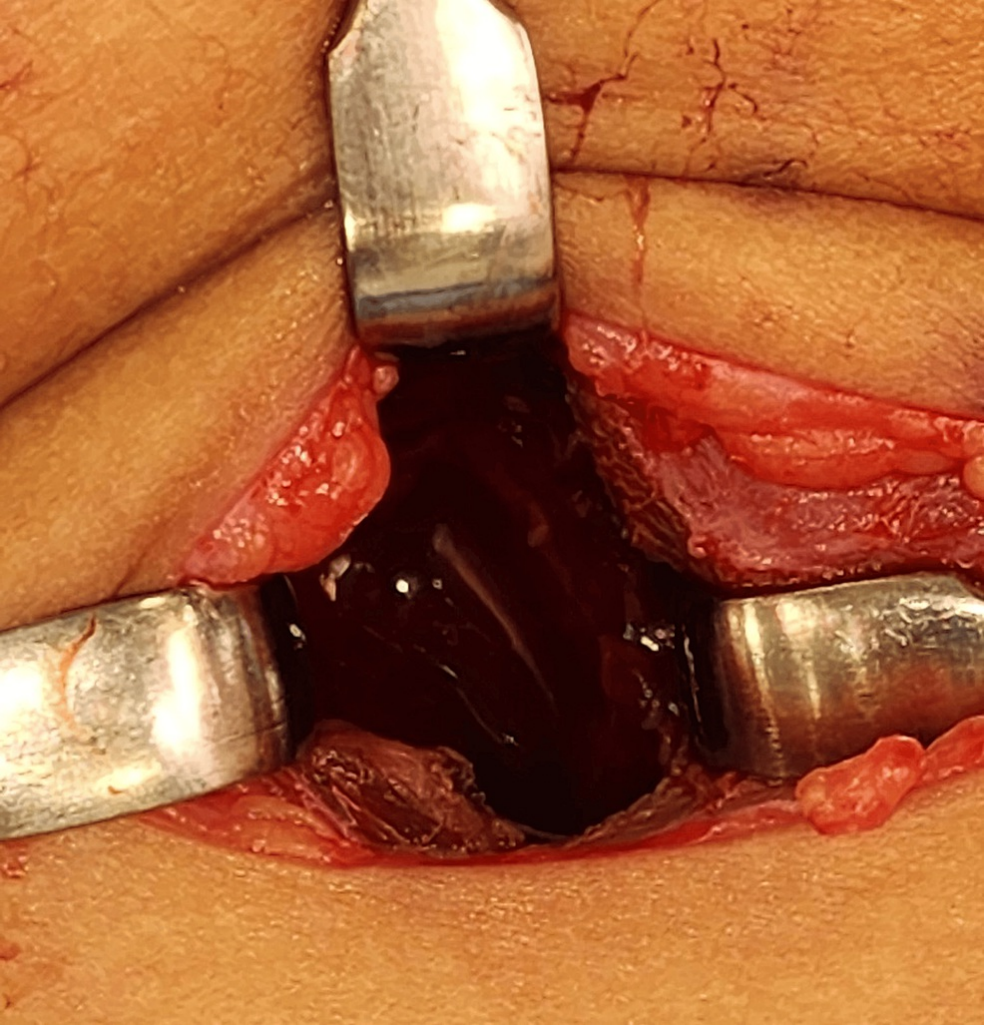
Perioperative photograph following abscess evacuation in the left upper thyroid lobe

During surgery, a 5-cm transcervical incision was made along a skin crease (Figure 3). The strap muscles were retracted to expose the upper lobe of the left thyroid gland. An aspiration using a 16-gauge needle yielded approximately 7cc of pus (Figure 4). A capsule incision and cavity suction were performed without intraoperative nerve monitoring due to the superficial location of the abscess. The cavity was flushed with diluted povidone (Figure 5), and a Penrose drain was inserted to aid wound management. Postoperatively, the child received intravenous amoxicillin/clavulanic acid and analgesics. The pus was cultured for sensitivity and tested for *Mycobacterium tuberculosis* using a polymerase chain reaction.

On postoperative day two, the Penrose drain was removed. The child was active, tolerating oral intake well, and experienced minimal neck pain. She was discharged on day three with oral antibiotics and analgesics to continue for one week. At discharge, the neck wound showed no signs of swelling or discharge and appeared clean.

## Discussion

Thyroid abscesses or acute suppurative thyroiditis (AST) characterized by abscess formation represent a rare condition, accounting for less than 1% of all thyroid diseases [5]. This condition typically arises from an infection within the thyroid gland that progresses to an abscess. In children, AST is often linked to congenital anomalies stemming from the third or fourth brachial arches [6] or a pyriform sinus fistula [7]. In adults, factors contributing to AST include thyroid cancer [8], large goiters [9], autoimmune thyroid diseases like Hashimoto's disease [10], and, occasionally, lymphatic or hematogenous spread, or iatrogenic causes following a fine needle aspiration biopsy [11]. Only 8% of AST cases occur in adults [12].

The patient presented with a painful swelling in the anterior neck region, persisting for two weeks, accompanied by odynophagia, low-grade fever, and leukocytosis, but without hyperthyroidism symptoms, despite the thyroid infection. Previous research suggests hyperthyroidism is not commonly associated with AST [13]. It is crucial to distinguish AST from subacute thyroiditis (SAT) due to significant differences in their management [14]. SAT typically responds well to corticosteroids [15].

Imaging studies, such as ultrasound and CT, are essential for accurately diagnosing and differentiating thyroid abscesses from other conditions, guiding treatment, and preventing complications. Ultrasound can differentiate between solid and cystic masses, while CT can pinpoint the abscess's exact location and its extension into deep neck spaces, facilitating surgical planning. CT can also detect a pyriform fistula [3], which requires further investigation after the acute infection phase, possibly through a barium swallow or endoscopic hypopharyngoscopy [16].

Effective management of a thyroid abscess involves draining the abscess, as demonstrated in this case report. Imaging aids in identifying the affected areas and planning the surgical approach and extent. Although initial treatments may include single or multiple needle aspirations with antibiotic coverage, surgery is often ultimately required. Common surgical procedures include incision and drainage, partial thyroidectomy, and, if a fistula tract is present, its excision or even total thyroidectomy [17]. Delaying treatment of a thyroid abscess can lead to severe complications, including mediastinitis, pericarditis, fistula formation [18], and even death [17].

## Conclusions

We report a case of a 5-year-old girl diagnosed with a multiloculated abscess within the left thyroid lobe. The patient was treated with successful incision and drainage, supported by antibiotic therapy, resulting in a full recovery without complications. This case underscores the importance of promptly diagnosing and treating thyroid abscesses to prevent significant morbidity and mortality. Despite its rarity, AST should be considered in the differential diagnosis of general neck swelling. Incorporating imaging techniques like ultrasound or CT in the evaluation process facilitates timely diagnosis and treatment, ensuring optimal patient outcomes.
